# An alternative pathway for the effective production of the omega‐3 long‐chain polyunsaturates EPA and ETA in transgenic oilseeds

**DOI:** 10.1111/pbi.12328

**Published:** 2015-01-30

**Authors:** Noemi Ruiz‐Lopez, Richard P. Haslam, Sarah Usher, Johnathan A. Napier, Olga Sayanova

**Affiliations:** ^1^ Department of Biological Chemistry and Crop ProtectionRothamsted ResearchHarpendenUK; ^2^ Instituto de la Grasa (CSIC)SevilleSpain

**Keywords:** metabolic engineering, lipids, omega‐3 polyunsaturated fatty acids, transgenic, *Camelina*

## Abstract

The synthesis and accumulation of omega‐3 long‐chain polyunsaturated fatty acids in transgenic *Camelina sativa* is demonstrated using the so‐called alternative pathway. This aerobic pathway is found in a small number of taxonomically unrelated unicellular organisms and utilizes a C18 Δ9‐elongase to generate C20 PUFAs. Here, we evaluated four different combinations of seed‐specific transgene‐derived activities to systematically determine the potential of this pathway to direct the synthesis of eicosapentaenoic acid (EPA) in transgenic plants. The accumulation of EPA and the related omega‐3 LC‐PUFA eicosatetraenoic acid (ETA) was observed up to 26.4% of total seed fatty acids, of which ETA was 9.5%. Seed oils such as these not only represent an additional source of EPA, but also an entirely new source of the *bona fide* fish oil ETA. Detailed lipidomic analysis of the alternative pathway in *Camelina* revealed that the acyl‐substrate preferences of the different activities in the pathway can still generate a substrate‐dichotomy bottleneck, largely due to inefficient acyl‐exchange from phospholipids into the acyl‐CoA pool. However, significant levels of EPA and ETA were detected in the triacylglycerols of transgenic seeds, confirming the channelling of these fatty acids into this storage lipid.

## Introduction

**Figure 1:**
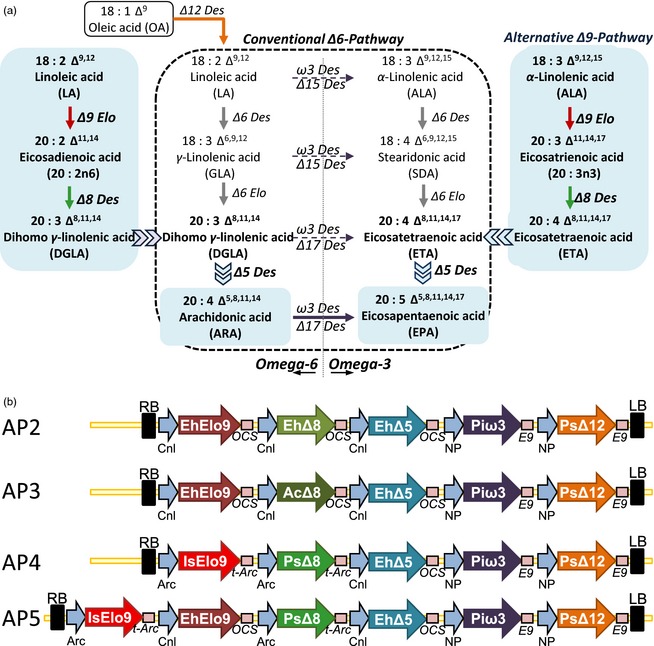
(a) Schematic representation of Δ6‐conventional and Δ9‐alternative pathways for LC‐PUFAs biosynthesis, with the different enzyme activities shown as arrows in different colours, also reflected in (b). (b) Simplified maps of vectors used for transformation of *Arabidopsis thaliana* and *Camelina sativa*. Cnl, conlinin 1 promoter; NP, napin promoter; Nos, nopaline synthase promoter; Arc, *P. vulgaris* promoter; EhElo9, a Δ9‐elongase from *E. huxleyi;* IsoElo9, a Δ9‐elongase from *I. galbana;* AcΔ8, a Δ8‐desaturase from *A. castellanii;* EhΔ8, a Δ8‐desaturase from *E. huxleyi;* PsΔ8, a Δ8‐desaturase from *P. Salina*; EhΔ5, Δ5‐desaturase from *E. huxleyi*; Piω3, *a* ω3‐desaturase from *P. infestans*; PsΔ12, a Δ12‐desaturase from *P. sojae*; and NTPII, neomycin phosphotransferase gene. OCS, E9, t‐Nos and t‐Arc represent terminators.

It is now well accepted that omega‐3 long‐chain polyunsaturated fatty acids (LC‐PUFAs), especially eicosapentaenoic acid (EPA; 20:5Δ^5,8,11,14,17^) and docosahexaenoic acid (DHA; 22:6Δ^4,7,10,13,16,19^), are vital for human health and nutrition and play a crucial role in preventing cardiovascular diseases and associated precursor conditions such as metabolic syndrome and obesity (Swanson *et al*., 2012). Currently, oily marine fish is the major dietary source of these fatty acids; however, considering growing pressure on global fish stocks and pollution of the marine environment, there is an urgent need for an alternative cost‐effective solution for large‐scale production of LC‐PUFAs. In recent years, the feasibility of using higher plants for the production of omega‐3 LC‐PUFAs has been explored, and considerable progress has been made for effective production of EPA and DHA in oilseeds (Petrie *et al*., 2012a,b; Ruiz‐Lopez *et al*., 2013, 2014). A variety of strategies have been used to introduce (via genetic engineering) the omega‐3 LC‐PUFAs metabolic pathways in oil crops, mainly by expressing desaturase and elongase genes involved in different biosynthetic routes for EPA and DHA accumulation (reviewed in Haslam *et al*., 2013). There are a number of different configurations of the biosynthetic pathway for C20 LC‐PUFAs production. In particular, two separate and converging aerobic routes exist for the biosynthesis of EPA and its omega‐6 counterpart, arachidonic acid (ARA; 20:4Δ^5,8,11,14^). The predominant sequence (commonly called the ‘conventional’ or ‘Δ6‐pathway’) of enzymatic reactions required to convert C18 fatty acids to C20+ PUFAs commences with the introduction of a double bond at the Δ6 position, followed by C2 chain elongation and a second desaturation at the Δ5 position in the C20 acyl chain, generating EPA from α‐linolenic acid (ALA; 18:3Δ^9,12,15^; *n*‐3) and generating ARA from linoleic acid (LA; 18:2Δ^9,12^; *n*‐6). In some species of microalgae such as *Pavlova salina* and *Isochrysis galbana* (Prymnesiophyceae), and protists such as *Euglena gracilis* (Euglenophyceae), *Acanthamoeba castellanii* and *Perkinsus marinus,* an alternative configuration for the synthesis of ARA and EPA (commonly known as the ‘alternative’ or ‘Δ9‐pathway’) has been observed (Damude *et al*., 2007; Korn, 1964; Sayanova *et al*., 2006a,b; Venegas‐Calerón *et al*., 2007; Wallis and Browse, 1999; Zhou *et al*., 2007). This pathway commences with the Δ9‐elongation by two carbons of LA and ALA, to yield eicosadienoic acid (20:2Δ^11,14^; 20:2*n*‐6) and eicosatrienoic acid (20:3Δ^11,14,17^; 20:3*n*‐3), respectively. These two fatty acids are then chain‐desaturated via a specific Δ8‐desaturase to generate dihomo‐γ‐linolenic acid (DGLA; 20:3Δ^8,11,14^; *n*‐6) and eicosatetraenoic acid (ETA; 20:4Δ^8,11,14,17^; *n*‐3). As in the prevalent Δ6‐pathway, these fatty acids are desaturated to ARA and EPA by a Δ5‐desaturase (see Figure 1a for a simplified representation of biosynthetic pathways).

One advance in increasing the accumulation of omega‐3 LC‐PUFAs in transgenic plants has been derived from the characterization and deployment of acyl‐CoA‐dependent desaturases (Sayanova *et al*., 2011a), which bypass the well‐documented substrate‐dichotomy bottleneck (a result of the differing acyl carrier substrate preferences of desaturases and elongases). One of the potential benefits of expressing the alternative Δ9‐pathway in seeds is to mitigate this acyl‐exchange requirement. The first C18 Δ9‐elongase, from *I. galbana*, was described by Qi *et al*. (2002), and subsequently, these authors went on to express the entire alternative Δ9‐pathway in *Arabidopsis thaliana* leaves (Qi *et al*., 2004), where plants were sequentially transformed with genes expressing a Δ9‐elongase, a Δ8‐desaturase from the protist *E. gracilis* and a fungal Δ5‐desaturase from *Mortierella alpina*; all under the control of the constitutive 35S viral promoter. The accumulation of 6.6% ARA and 3.0% EPA in total lipids of leaf tissues represented a ‘proof‐of‐concept’ demonstration for the plant synthesis of LC‐PUFAs and also for the functionality of the alternative Δ9‐pathway in plants. However, the choice of the constitutive 35S promoter limited the expression of the transgenes to vegetative tissues, and detailed analyses of leaf lipids indicated an inefficient transfer of these nonnative fatty acids from the acyl‐CoA pool into extraplastidial phospholipids (Sayanova *et al*., 2006a). Other examples of the utilization of the alternative Δ9‐pathway for LC‐PUFAs synthesis have been reported by Petrie *et al*. (2010) where three Δ9‐elongases from *Emiliania huxleyi*,* Pavlova pinguis* and *P. salina* were jointly expressed in the leaves of *Nicotiana benthamiana*, generating total lipids containing 1.2% EPA and 0.7% DHA. Subsequently, the seed‐specific expression of the alternative Δ9‐pathway (*I. galbana* Δ9‐elongase and *P. salina* Δ8‐ and Δ5‐desaturases) in Arabidopsis produced up to 20% ARA and 2% EPA in seed oil. This pathway was also transformed in *Brassica napus* where it yielded approximately 10% ARA and 2% EPA in seed oil, and the majority of these omega‐6 fatty acids were located at the *sn*‐2 position of triacylglycerol, TAG (Petrie *et al*., 2012a,b).

In this study, we build on those earlier observations to optimize the reconstitution of omega‐3 EPA biosynthesis in transgenic *Arabidopsis thaliana* and *Camelina sativa* seeds exclusively via the alternative Δ9‐pathway. We also discuss the advantages/disadvantages of using this Δ9‐pathway instead of expressing an acyl‐CoA‐dependent Δ6‐desaturase activity due to the circumvention of the well‐documented ‘substrate‐dichotomy’ bottleneck.

## Results and discussion

### Reconstitution of the alternative Δ9‐pathway in transgenic Arabidopsis seeds

In previous studies, we investigated the effects of different combinations of genes and promoters on the accumulation of *n*‐3 LC‐PUFAs in Arabidopsis and *C. sativa* using the conventional Δ6‐pathway (Ruiz‐Lopez *et al*., 2009, 2013, 2014; Ruiz‐López *et al*., 2012; Sayanova *et al*., 2011a) . We demonstrated the advantages of utilizing an acyl‐CoA‐dependent Δ6‐desaturase such as that reported from *Ostreococcus tauri* instead of phospholipid‐dependent forms of this enzyme activity (Domergue *et al*., 2003, 2005). This was ascribed to the circumvention of the first (and potentially rate‐limiting) acyl‐exchange bottleneck between phospholipids and the acyl‐CoA pool.

Looking for other alternatives to bypass this generic metabolic bottleneck and to further optimize the production of EPA in oilseed crops such as *C. sativa*, we systematically generated a set of constructs carrying different genes encoding the enzymes participating in the alternative Δ9‐pathway (Δ9‐elongating activity and Δ8‐ and Δ5‐desaturases) for the formation of C20 polyunsaturated fatty acids. We also included other additional genes encoding an omega‐3 desaturase and a Δ12‐desaturase, to optimize the production of EPA in seeds and also to allow a direct comparison with analogous studies in Arabidopsis and *C. sativa* using the Δ6‐pathway containing these additional activities (Ruiz‐Lopez *et al*., 2013, 2014). These constructs were initially evaluated in Arabidopsis, and after transformation via floral dip, mature seeds from Kan^R^‐plants were analysed by GC–MS for total fatty acid composition (see Figure 1b for schematic representation of constructs).

The first two constructs (designated AP2 and AP3), contained the minimal three genes required for the synthesis of *n*‐3/*n*‐6 C20 LC‐PUFAs (e.g. ARA and EPA) from endogenous C18 substrates plus a Δ12‐desaturase gene from *Phytophthora sojae* (Ps∆12) to enhance the levels of LA, and a C20 omega‐3 desaturase from *Phytophthora infestans* (Piω3; Wu *et al*., 2005; Ruiz‐López *et al*., 2012) to increase the conversion of ARA to EPA (Figure 1a). Specifically, the AP2 construct contained a ∆9‐fatty acid elongase gene (EhElo9), a ∆8‐ and a ∆5‐desaturase genes (Eh∆8 and EhΔ5), all isolated from the coccolithophore *E. huxleyi* (Sayanova *et al*., 2011b), whereas in construct AP3, the ∆8‐desaturase gene Eh∆8 from *E. huxleyi* was replaced by a ∆8‐desaturase gene Ac∆8 from *A. castellanii* (Sayanova *et al*., 2006a) (Figure 1b). In the case of the EhΔ5 ∆5‐desaturase, this is believed to have a preference for acyl‐CoA‐substrates (Sayanova *et al*., 2011b), whereas all the other desaturases used in this study are assumed to utilize phospholipid‐linked fatty acids as their substrates.

Analysis of total fatty acid methyl esters (FAMEs) from the seeds of 28 and 38 individual transgenic Arabidopsis T2 lines for AP2 and AP3 constructs, respectively, indicated that plants expressing these genes accumulated significant levels of 20:2*n*‐6 (7.9% and 6.7%, respectively) and 20:3*n*‐3 (4.9% and 4.8%, respectively), as a result of LA and ALA elongation by EhElo9 (as previously observed in yeast by Sayanova *et al*., 2011b). Disappointedly, EhΔ8 and AcΔ8 seemed not to be active in Arabidopsis as no accumulation of Δ8‐desaturation products nor EPA was detected (Table 1), although both genes were previously shown by us to be active in yeast. PCR demonstrated the presence of transcripts in Arabidopsis of the expected size for both desaturases, although no data were generated to assess the presence of encoded polypeptides.

**Table 1:** FAMEs (mol%) of total lipids in *A. thaliana* and *C. sativa* mature seeds

	*A. thaliana*					*C. sativa*		
	WT	AP2	AP3	AP4	AP5	WT	AP5	AP5#1
16:0	6.4 ± 0.2	6.1 ± 0.4	6.3 ± 0.4	9.7 ± 0.4	11.0 ± 0.9	7.2 ± 0.2	10.9 ± 2.5	7.4 ± 0.3
18:0	3.2 ± 0.1	3.2 ± 0.2	3.2 ± 0.1	3.6 ± 0.1	3.7 ± 0.2	3.4 ± 0.2	4.2 ± 0.6	3.9 ± 0.3
18:1n9 (OA)	14.6 ± 1.0	11.3 ± 1.4	10.9 ± 1,4	11.9 ± 1.4	11.9 ± 1.5	11.5 ± 1.6	9.2 ± 2.0	9.7 ± 1.3
18:1n7	1.8 ± 0.2	1.8 ± 0.2	1.9 ± 0.3	1.9 ± 0.2	1.8 ± 0.3	0.8 ± 0.0	1.1 ± 0.2	0.8 ± 0.1
18:2n6 (LA)	28.2 ± 0.7	26.3 ± 1.3	26.4 ± 1.7	28.7 ± 0.7	26.2 ± 1.2	18.6 ± 0.6	19.4 ± 2.2	15.3 ± 1.1
18:3n3 (ALA)	15.6 ± 0.6	13.6 ± 1.0	14.6 ± 1.3	12.9 ± 0.8	12.6 ± 0.7	34.6 ± 1.0	21.8 ± 3.2	24.5 ± 2.8
20:0	2.3 ± 0.2	2.2 ± 0.2	2.2 ± 0.1	2.0 ± 0.1	1.6 ± 0.1	1.9 ± 0.2	1.7 ± 0.2	1.7 ± 0.1
20:1n9	19.9 ± 0.8	16.7 ± 1.1	16.5 ± 1.1	10.7 ± 1.3	8.8 ± 1.2	13.4 ± 0.3	5.8 ± 1.7	5.7 ± 2.0
20:1n7	2.5 ± 0.2	2.4 ± 0.4	2.4 ± 0.5	1.8 ± 0.4	1.4 ± 0.2	0.7 ± 0.0	0.5 ± 0.1	0.4 ± 0.1
20:2n9	–	–	–	3.0 ± 0.8	3.3 ± 0.8	–	2.6 ± 0.4	4.1 ± 1.2
20:*2n6*	2.1 ± 0.2	7.9 ± 1.9	6.7 ± 2.1	1.7 ± 1.1	1.9 ± 0.9	2.2 ± 0.1	1.7 ± 0.6	1.2 ± 0.4
20:3n6 (DGLA)	–	–	–	2.9 ± 1.1	2.6 ± 0.8	–	4.8 ± 2.3	2.8 ± 0.4
20:3n3	0.5 ± 0.1	4.9 ± 1.5	4.8 ± 1.8	1.0 ± 0.7	1.5 ± 0.9	1.6 ± 0.1	2.0 ± 0.8	1.9 ± 0.2
20:4n6 (ARA)	–	–	–	0.6 ± 0.3	0.6 ± 0.2	–	1.3 ± 0.5	1.4 ± 0.4
20:4n3 (ETA)	–	–	–	3.3 ± 1.2	3.9 ± 0.9	–	5.8 ± 1.7	7.1 ± 1.5
20:5n3 (EPA)	–	–	–	1.9 ± 1.3	3.6 ± 1.2	–	4.7 ± 1.5	8.8 ± 3.5
22:0	0.4 ± 0.1	0.4 ± 0.1	0.4 ± 0.1	0.4 ± 0.1	0.4 ± 0.1	0.4 ± 0.0	0.3 ± 0.1	0.3 ± 0.0
22:1n9	2.0 ± 0.2	1.6 ± 0.2	1.7 ± 0.2	1.0 ± 0.2	0.6 ± 0.1	2.6 ± 0.2	0.8 ± 0.2	0.8 ± 0.4
Others	0.6 ± 0.1	1.8 ± 0.4	1.9 ± 0.5	1.1 ± 0.2	2.4 ± 1.0	1.2 ± 0.1	1.3 ± 0.4	2.2 ± 0.2
		T2 *n* = 28	T2 *n* = 38	T2 *n* = 14	T2 *n* = 35		T2 *n* = 8	T3 *n* = 19

Others include 16:1, 16:2, 24:0 and 24:1.

Tx, Generation; *n*, number of individual plants; *A*. *thaliana*,* Arabidopsis thaliana*;* C*. *sativa*,* Camelina sativa;* OA, oleic acid; LA, linoleic acid; ALA, α‐linolenic acid; DGLA—dihomo‐γ‐linolenic acid; ARA, arachidonic acid; ETA, eicosatetraenoic acid; EPA—eicosapentaenoic acid.

In an attempt to improve the conversion of LA and ALA fatty acids into the target C20 omega‐3 LC‐PUFA EPA, a third construct, AP4, was designed (Figure 1b). Previous studies have indicated that the expression of the *I. galbana* C18 Δ9‐polyunsaturated fatty acid specific elongase (IsoELO9, Qi *et al*., 2002, 2004) resulted in the accumulation of 20:2*n*‐6 and 20:3*n*‐3 in yeast and Arabidopsis. Additionally, the AP4 construct contained a *P. salina* Δ8‐desaturase, plus the EhΔ5, Piω3‐ and PsΔ12‐desaturases as in AP2 and AP3. Fatty acid analysis of T2 seeds from 14 individual transgenic lines for this construct revealed that there was some accumulation of the target omega‐3 LC‐PUFAs, EPA and ETA (averaged 1.9% and 3.3%, respectively). We also observed that the levels of the intermediates 20:2*n‐*6 and 20:3*n‐*3 were similar to WT, indicating that PsΔ8 was active. Additionally, AP4 seeds accumulated 20:2Δ^8,11^ (20:2*n*‐9, averaged level 3.0%), most likely generated as a side reaction of the PsΔ8 acting on endogenous 20:1Δ^11^ (20:1*n*‐9)—notably, the levels of this fatty acid were reduced in AP4 compared with WT (Table 1).

Taking into account the data provided by these iterations, a further six‐gene construct designated AP5 was generated by combining the most active genes present in AP2, AP3 and AP4 (EhELO9, IgElo9, PsΔ8, EhΔ5; Figure 1b). T2 transgenic Arabidopsis lines (*n* = 35) for AP5 were generated, and EPA was seen to accumulate to an average level of 3.6% (in the range 1.7–6.0% of total seed fatty acids; Table 1). Moreover, the levels of the both intermediates 20:2*n‐*6 and 20:3*n‐*3 were similar to the values found in WT, confirming our previous observations of PsΔ8 working very efficiently. However, some minor accumulation of Δ8‐desaturated fatty acids, 20:3*n‐6* (DGLA) and ETA, was also observed (an average of 2.6% and 3.9%, respectively) in T2 seeds (Table 1). This could be an indication that EhΔ5 was not as efficient as might be predicted from previous heterologous expression in yeast (Sayanova *et al*., 2011b). However, we believe that this is more likely a reflection of the substrate preference of this particular enzyme (for acyl‐CoA as opposed to phospholipids), which in turn replicated the problem of substrate dichotomy observed in the Δ6‐conventional pathway, as the preceding step (Δ8‐desaturation) most likely occurs on phospholipids (Figure 1a). As expected, there was some accumulation of 20:2*n*‐9, as in AP4 (via off‐target Δ8‐desaturation) and the levels of LA and ALA in AP5 transgenic seeds showed some minor reduction, compared with WT (Table 1). Finally, the levels of newly synthesized C20 omega‐6 fatty acids (DGLA and ARA; 2.6% and 0.6%, respectively) were much lower than the analogous omega‐3 forms (ETA and EPA; 3.9% and 3.6%, respectively). Notably, this is significantly different to the fatty acid profile reported by Petrie *et al*. (2012a,b), in which the expression of IsoELO9, PsΔ8 and PsΔ5 in transgenic Arabidopsis seeds resulted in higher levels of omega‐6 fatty acids such as ARA compared with EPA and ETA. However, the observed difference is likely due to the *fae1/fad3* mutant background used by Petrie *et al*. in the 2012 study, and also the absence of any omega‐3 desaturase in their construct.

### Reconstitution of the alternative Δ9‐pathway in *Camelina* seeds

In view of these results, the AP5 construct was introduced into *C. sativa* plants via Agrobacterium‐mediated floral transformation. The efficacy of *C. sativa* as a host for the biosynthesis of omega‐3 LC‐PUFAs has previously been demonstrated for the expression of the conventional Δ6‐pathway (Petrie *et al*., 2014; Ruiz‐Lopez *et al*., 2014); success that largely results from the high endogenous levels of ALA in seeds, the starting substrate for the omega‐3 biosynthetic pathway (Figure 1a). Moreover, the commonality of host, regulatory elements and transformation methods permitted the systematic comparison of the Δ6‐pathway with the alternative Δ9‐pathway. Analysis of total FAMEs from the seeds of eight individual transgenic *C. sativa* T2 lines expressing the AP5 construct demonstrated the accumulation of EPA. The mean levels of EPA found in these T2 lines ranged from 1.4 to 6.3% of total fatty acids in mature seeds (mean 4.7%; Table 1). Accumulation of nonnative fatty acids was further monitored in the T3 generation derived from selected T2 plants (Table 1). Mean EPA levels in T3 seeds increased on average from 4.7 to 8.8% in AP5 plants. The highest EPA value observed in an individual T3 line (AP5#1_5) was 14.8%. Further analyses demonstrated that the highest EPA value observed in an individual seed of AP5#1_5 was 16.9% of total fatty acids (see Figure S2; Table S1). Compared to our previous results obtained with the expression of the Δ6‐pathway in Camelina, the current levels of EPA are roughly two‐thirds of that seen with the conventional pathway (14.8% versus 23.9%; cf. Ruiz‐Lopez *et al*., 2014). However, as discussed below, the C20 omega‐3 LC‐PUFA ETA also accumulated to significant levels, contributing to the total levels of omega‐3 fish oils.

Interestingly, it was previously observed in transgenic Camelina lines expressing the *O. tauri* Δ6‐desaturase from the conventional pathway that the amount of oleic acid (18:1Δ^9^; OA; *n*‐9) decreased dramatically from 14.5% in the WT to <6% (Ruiz‐Lopez *et al*., 2014; Sayanova *et al*., 2011a). However, a similar pattern was not observed with the AP5 alternative pathway configuration, with levels of OA only slightly reduced (11.5% in WT versus 9.2% and 9.7% in T2 and T3 AP5 seeds). The most logical explanation for these combined observations is that the acyl‐CoA‐dependent *O. tauri* Δ6‐desaturase used in the conventional pathway is generating a strong pull on the OA‐CoA pool, which in conjunction with the oomycete Δ12‐desaturase (PsΔ12) generates the resulting depression of OA levels. However, it is clear that the presence of the PsΔ12 activity itself does not significantly reduce OA levels, as it is present in the AP5 construct (Figure 1a), and converts OA to LA. Thus, the alternative pathway represents a superior configuration of activities to avoid the reduction of OA.

We also do not observe a significant reduction in the levels of the omega‐6 precursor LA. The levels were 18.6% in WT, 19.4% in T2 AP5 seeds and 15.3% in T3 AP5 transgenic seeds. Overall, the levels of newly synthesized omega‐3 fatty acids were higher than those of omega‐6 acids, in T3 Camelina seeds. In contrast, expression of the alternative pathway genes had a clear effect on both ALA and 20:1*n*‐9 endogenous fatty acids. In particular, T2 seeds of AP5 contained a substantially decreased ALA content (from an average of 34.6% in WT to an average of 21.8% and 24.5% in T2 and T3 transgenic seeds, respectively; Table 1). All these observations (no impact in OA and LA levels, decreased levels of ALA, plus the fact that there are more omega‐3 fatty acids newly synthetized than omega‐6 ones) imply that the EhElo9 and IgElo9 have a substrate preference for omega‐3 ALA over omega‐6 LA. However, this (likely) situation is not directly confirmed, as the presence of the Piω3‐desaturase (which converts C20 omega‐6 fatty acids to omega‐3 forms) can potentially confound such a conclusion—thus, formal confirmation of the substrate preference of these enzymes needs to still be evaluated. In addition, as mentioned above, the 20:1*n*‐9 content was markedly decreased in AP5 lines, from the average levels of 13.8% in WT, to 5.9% in transgenic lines. This is most likely the consequence of off‐target PsΔ8‐desaturation and the generation of 20:2*n*‐9.

### Lipid profiling of the alternative Δ9‐pathway line AP5—comparison with the conventional Δ6‐pathway

To further characterize the accumulation of nonnative LC‐PUFAs in transgenic *C. sativa*, a more detailed lipid class analysis was performed on the T3 seeds of line AP5_1_5 (EPA content averaged 14.4%). Analyses of neutral and polar lipids revealed that EPA was almost equally distributed between neutral lipids (14.4%) and phospholipids lipids (12.4%), and EPA level was lower in glycolipid fraction (averaged 8.9%) (see Table 2). This pattern of partitioning is similar to our previous observations in Camelina seeds expressing the conventional pathway (Ruiz‐Lopez *et al*., 2014), but slightly reversed compared with Arabidopsis seeds (Ruiz‐López *et al*., 2012). In contrast, ETA was asymmetrically distributed between neutral lipids and phospholipid fraction. The phospholipid fraction was significantly enriched in this fatty acid (17.6%) in comparison with the levels found in neutral lipids (9.1%; Table 2). The most likely explanation for this phenomenon is that the precursor 20:3*n‐*3 is efficiently desaturated by the PsΔ8‐desaturase to generate ETA and that this reaction occurs in the phospholipid pool (on account of the above‐noted substrate preference of PsΔ8). However, the subsequent Δ5‐desaturation (via EhΔ5) is impeded by the substrate dichotomy generated by the acyl‐CoA‐dependent nature of this latter enzyme. It should be noted that the identity of ETA as 20:4Δ^8,11,14,17^ was based on the fact that it did not comigrate with authentic standards for other 20:4 fatty acids of a similar m/z (304.24 neutral ion) such as arachidonic acid (20:4Δ^5,8,11,14^) and the nonmethylene‐interrupted fatty acid juniperonic acid (20:4Δ^5,11,14,17^). In the latter case, this was demonstrated by using verified biological standards previously generated in transgenic Arabidopsis lines accumulating juniperonic acid (Figure S1; Sayanova *et al*., 2007). Interestingly, ETA is often observed at low levels in fish oils and has been ascribed anti‐inflammatory activities similar to EPA (Ghioni *et al*., 2004). Thus, ETA can be considered as an additional omega‐3 LC‐PUFA widely distributed in marine food webs.

**Table 2:** Fatty acid composition (Mol%) of main lipid classes. Line AP5#1_5 (T3)

	Total lipids		Neutral lipids		Phospholipids		Glycolipids	
	WT	AP5#1_5	WT	AP5#1_5	WT	AP5#1_5	WT	AP5#1_5
16:0	6.8 ± 0.3	7.9 ± 0.1	6.8 ± 0.2	6.9 ± 0.1	13.3 ± 0.4	20.5 ± 0.9	16.8 ± 1.5	19.0 ± 2.6
18:0	3.1 ± 0.0	4.4 ± 0.0	3.5 ± 0.3	4.3 ± 0.1	3.5 ± 1.0	5.4 ± 0.5	8.8 ± 1.4	10.3 ± 1.3
18:1n9 (OA)	16.1 ± 0.7	8.2 ± 0.1	15.2 ± 0.7	8.4 ± 0.1	35.8 ± 2.6	4.0 ± 0.3	13.9 ± 0.7	6.9 ± 0.5
18:1n7	0.8 ± 0.0	0.6 ± 0.1	0.9 ± 0.1	0.6 ± 0.1	0.9 ± 0.7	1.5 ± 0.0	0.8 ± 0.0	0.9 ± 0.1
18:2n6 (LA)	18.1 ± 0.6	13.0 ± 0.1	18.4 ± 0.5	13.1 ± 0.1	28.2 ± 0.9	14.5 ± 0.1	14.9 ± 1.5	11.6 ± 0.6
18:3n3 (ALA)	32.7 ± 0.9	21.5 ± 0.2	31.4 ± 0.4	21.6 ± 0.3	13.0 ± 0.2	11.2 ± 0.4	21.7 ± 2.8	14.1 ± 1.7
20:0	1.8 ± 0.0	1.9 ± 0.0	2.0 ± 0.2	2.0 ± 0.0	0.3 ± 0.1	0.3 ± 0.1	1.7 ± 0.0	1.6 ± 0.2
20:1n9	14.0 ± 0.4	3.6 ± 0.0	14.2 ± 0.4	3.5 ± 0.2	2.6 ± 0.6	0.7 ± 0.1	8.9 ± 0.1	2.0 ± 0.4
20:1n7	0.5 ± 0.1	0.1 ± 0.0	0.6 ± 0.1	0.3 ± 0.1	0.1 ± 0.1	–	0.5 ± 0.2	0.2 ± 0.0
20:2n9	–	6.0 ± 0.0	–	6.2 ± 0.1	–	3.8 ± 0.1	–	3.8 ± 0.6
20:2n6	1.9 ± 0.1	0.8 ± 0.0	1.9 ± 0.1	0.7 ± 0.0	0.5 ± 0.3	0.8 ± 0.0	1.3 ± 0.1	0.8 ± 0.0
20:3n6 (DGLA)	–	2.9 ± 0.1	–	3.0 ± 0.1	–	2.2 ± 0.1	–	2.2 ± 0.2
20:3n3	1.3 ± 0.1	1.4 ± 0.0	1.2 ± 0.0	1.4 ± 0.1	0.3 ± 0.0	1.2 ± 0.1	0.8 ± 0.1	0.9 ± 0.2
20:4n6 (ARA)	–	1.7 ± 0.0	–	1.8 ± 0.1	–	0.4 ± 0.0	–	1.2 ± 0.1
20:4n3 (ETA)	–	9.2 ± 0.1	–	9.1 ± 0.5	–	17.6 ± 0.9	–	5.9 ± 0.5
20:5n3 (EPA)	–	14.5 ± 0.2	–	14.4 ± 0.3	–	12.4 ± 0.5	–	8.9 ± 1.0
22:0	0.3 ± 0.0	0.3 ± 0.0	0.4 ± 0.0	0.4 ± 0.0	–	–	0.5 ± 0.0	0.4 ± 0.0
22:1n9	2.5 ± 0.1	0.4 ± 0.0	2.6 ± 0.2	0.4 ± 0.0	0.2 ± 0.1	–	1.8 ± 0.0	0.3 ± 0.0
Others	0.8 ± 0.0	2.3 ± 0.1	3.8 ± 0.3	2.5 ± 0.1	1.5 ± 0.2	3.3 ± 0.6	10.0 ± 2.5	9.9 ± 1.9

Others include 16:1, 16:2, 24:0 and 24:1.

OA, Oleic acid; LA, linoleic acid; ALA, α‐linolenic acid; DGLA—dihomo‐γ‐linolenic acid; ARA, arachidonic acid; ETA, eicosatetraenoic acid; EPA—eicosapentaenoic acid.

**Figure 2:**
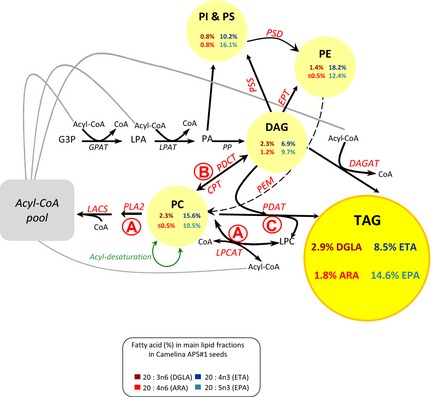
Schematic diagram of the main lipid classes and biochemical pathways involved in the production of TAG in developing seeds. Indicated are the omega‐3 and omega‐6 LC‐PUFAs percentages (%) in main lipid fractions of *Camelina sativa* seeds from line AP5#1_5 (T3). The relative sizes of the different pools are not directly reflected by the size of each node—the predominant major lipid class is TAG, followed by DAG. The major phospholipid species is PC, and then PE, although all phospholipids are minor components compared to neutral lipids. The acyl‐CoA pool is a very small but highly dynamic pool. Fluxes between the different pools are bidirectional (apart from the terminal synthesis of TAG) and consequently can only be inferred from these steady‐state measurements. CPT, CDP‐choline: diacylglycerol cholinephosphotransferase; DGAT, acyl‐CoA:diacylglycerol acyltransferase; EPT, CDP‐ethanolamine:diacylglycerol ethanolaminephosphotransferase; G3P, glycerol‐3‐phosphate; GPAT, acyl‐CoA:glycerol 3‐phosphate acyltransferase; LACS, long‐chain acyl‐CoA synthetase; LPA, lysophosphatidic acid; LPAT, acyl‐CoA:lysophosphatidic acid acyltransferase; LPCAT, acyl‐CoA:lysophosphatidylcholine acyltransferase; PA, phosphatidic acid; PAP, phosphatidic acid phosphatase; PDAT, phospholipid:diacylglycerol acyltransferase; PDCT, phosphatidylcholine:diacyglycerol cholinephosphotransferase; PEM, phosphatidylethanolamine N‐methyltransferase; PLA_2_, phospholipase A2; PSD, phosphatidylserine decarboxylase; and PSS, phosphatidylserine synthase.

To better understand the distribution of these nonnative fatty acids in *C. sativa* seeds, we performed a more detailed examination of the lipidome, using similar approaches as previously described for transgenic lines expressing the conventional Δ6‐pathway (Ruiz‐Lopez *et al*., 2014). Initially, examining the mid‐stage of seed development, the class distribution of EPA and other nonnative fatty acids was determined for AP5_1_5 line and compared to wild‐type *C. sativa* (Table 3 and Figure 2). Interestingly, EPA levels were greater in TAG (14.6%) than diacylglycerols (DAG; 9.7%). These observations are similar to those previously reported for Camelina seeds synthesizing EPA via the conventional pathway (Ruiz‐Lopez *et al*., 2014). It was also noted that the levels of 16:0 were markedly increased in DAG, but not TAG, whilst OA, LA and ALA levels were decreased in the neutral lipids of Camelina expressed conventional or alternative pathways. The possible causes for these alterations are discussed below in the context of wider glycerolipid metabolism.

**Table 3:** Fatty acid composition (mol%) of neutral lipid fraction. Line AP5#1_5 (T3)

	TAG		DAG		FFA	
	WT	AP5#1_5	WT	AP5#1_5	WT	AP5#1_5
16:0	7.0 ± 0.5	7.1 ± 0.2	12.0 ± 0.9	17.0 ± 1.1	10.1 ± 1.6	12.5 ± 0.9
18:0	3.4 ± 0.2	4.3 ± 0.1	8.4 ± 1.0	13.3 ± 1.2	6.4 ± 1.5	8.7 ± 0.7
18:1n9 (OA)	14.7 ± 1.4	8.5 ± 0.3	21.1 ± 2.3	7.7 ± 0.6	14.9 ± 1.8	7.5 ± 0.1
18:1n7	0.8 ± 0.0	0.6 ± 0.1	0.8 ± 0.0	0.8 ± 0.1	0.6 ± 0.3	0.1 ± 0.0
18:2n6 (LA)	17.9 ± 0.6	13.1 ± 0.2	16.7 ± 1.0	10.8 ± 0.8	14.5 ± 1.0	10.7 ± 0.3
18:3n3 (ALA)	32.6 ± 0.9	22.1 ± 0.3	21.5 ± 0.8	12.4 ± 1.5	30.3 ± 2.3	18.1 ± 0.5
20:0	1.9 ± 0.4	2.0 ± 0.0	1.8 ± 0.6	2.3 ± 0.4	2.2 ± 0.2	2.1 ± 0.1
20:1n9	14.3 ± 0.5	3.7 ± 0.2	9.2 ± 1.5	2.6 ± 0.3	11.8 ± 0.5	2.9 ± 0.4
20:1n7	0.4 ± 0.2	0.2 ± 0.1	0.4 ± 0.1	0.3 ± 0.1	0.5 ± 0.1	0.3 ± 0.1
20:2n9	–	6.0 ± 0.1	–	4.0 ± 0.4	–	5.2 ± 0.2
20:2n6	2.0 ± 0.2	0.7 ± 0.0	1.4 ± 0.2	0.9 ± 0.1	1.5 ± 0.1	0.6 ± 0.1
20:3n6 (DGLA)	–	2.9 ± 0.1	–	2.3 ± 0.2	–	2.6 ± 0.1
20:3n3	1.3 ± 0.1	1.4 ± 0.0	1.0 ± 0.2	1.2 ± 0.2	1.3 ± 0.1	1.2 ± 0.1
20:4n6 (ARA)	–	1.8 ± 0.0	–	1.2 ± 0.1	–	1.7 ± 0.2
20:4n3 (ETA)	–	8.5 ± 0.2	–	6.9 ± 0.6	–	7.6 ± 0.1
20:5n3 (EPA)	–	14.6 ± 0.4	–	9.7 ± 1.0	–	13.0 ± 0.5
22:0	0.3 ± 0.0	0.3 ± 0.0	0.9 ± 0.2	1.5 ± 0.3	0.9 ± 0.3	1.2 ± 0.2
22:1n9	2.5 ± 0.2	0.4 ± 0.0	1.8 ± 0.6	0.6 ± 0.2	2.3 ± 0.2	0.6 ± 0.5
Others	0.9 ± 0.1	1.9 ± 0.2	2.9 ± 0.6	4.7 ± 0.9	2.6 ± 0.7	3.2 ± 0.4

Others include 16:1, 16:2, 24:0 and 24:1.

OA, Oleic acid; LA, linoleic acid; ALA, α‐linolenic acid; DGLA—dihomo‐γ‐linolenic acid; ARA, arachidonic acid; ETA, eicosatetraenoic acid; EPA—eicosapentaenoic acid.

In view of these data, and to develop a more accurate hypothesis regarding the flux of these fatty acids through the different lipid classes, we also determined the acyl composition of the major phospholipids (Table 4 and Figure 2): phosphatidylcholine (PC), phosphatidylethanolamine (PE), phosphatidylinositol (PI) and phosphatidylserine (PS), as described before (Ruiz‐Lopez *et al*., 2014). In the case of EPA, in AP5_1_5, there was a similar level of accumulation in PC and PE phospholipid fractions (10.5% and 12.4%, respectively). However, EPA levels were increased in the fraction that contained both PI and PS (16.1%). Interestingly, ETA was asymmetrically distributed in the three phospholipid fractions, with higher ETA levels found in PE (18.2%) > PC (15.6%) > PI&PS (10.2%). Notably, the levels of ETA and EPA present in the PI&PS fraction are similar in ratio and to those in DAG, reflecting the biosynthesis of PI and PS from CDP_DAG (Figure 2), although the relative size of the phospholipid (PC, PE, PI&PS) pools is small compared with those for neutral lipids such as DAG and (the predominant) TAG.

**Table 4:** Fatty acid composition (mol%) of phospholipids in line AP5#1_5 (T3)

	PC		PE		PI&PS	
	WT	AP5#1_5	WT	AP5#1_5	WT	AP5#1_5
16:0	14.1 ± 1.3	21.0 ± 0.8	14.9 ± 1.2	23.4 ± 2.6	16.2 ± 1.1	22.9 ± 3.1
18:0	7.3 ± 1.7	10.1 ± 1.3	2.1 ± 0.3	6.6 ± 2.6	8.2 ± 0.3	8.5 ± 0.7
18:1n9 (OA)	39.0 ± 2.1	5.8 ± 1.4	29.0 ± 0.7	2.5 ± 0.1	24.6 ± 0.2	2.8 ± 1.7
18:1n7	0.0 ± 0.0	1.1 ± 0.0	0.0 ± 0.0	1.5 ± 0.1	0.0 ± 0.0	0.7 ± 0.0
18:2n6 (LA)	24.3 ± 1.0	12.5 ± 0.5	34.6 ± 1.4	15.0 ± 0.6	25.4 ± 0.2	13.3 ± 0.3
18:3n3 (ALA)	10.5 ± 0.4	10.0 ± 0.6	13.5 ± 0.6	10.9 ± 0.5	17.9 ± 0.0	13.7 ± 0.3
20:0	0.4 ± 0.0	0.5 ± 0.1	0.1 ± 0.1	0.3 ± 0.1	0.4 ± 0.0	0.6 ± 0.3
20:1n9	1.7 ± 0.1	0.8 ± 0.1	1.0 ± 0.1	0.2 ± 0.0	1.1 ± 0.1	0.4 ± 0.1
20:1n7	0.0 ± 0.0	0.1 ± 0.1	0.0 ± 0.0	0.0 ± 0.0	0.0 ± 0.0	0.0 ± 0.1
20:2n9	–	3.8 ± 0.2	–	2.7 ± 0.2	–	2.2 ± 0.4
20:2n6	0.5 ± 0.0	0.9 ± 0.0	0.3 ± 0.0	0.6 ± 0.0	0.3 ± 0.0	0.3 ± 0.0
20:3n6 (DGLA)	–	2.3 ± 0.1	–	1.4 ± 0.1	–	0.8 ± 0.1
20:3n3	0.3 ± 0.0	1.3 ± 0.0	0.1 ± 0.0	0.9 ± 0.2	0.2 ± 0.0	0.6 ± 0.1
20:4n6 (ARA)	–	0.3 ± 0.0	–	0.3 ± 0.1	–	0.8 ± 0.1
20:4n3 (ETA)	–	15.6 ± 1.2	–	18.2 ± 1.8	–	10.2 ± 0.8
20:5n3 (EPA)	–	10.5 ± 0.7	–	12.4 ± 1.4	–	16.1 ± 1.2
22:0	0.5 ± 0.1	0.7 ± 0.2	0.0 ± 0.0	0.1 ± 0.1	0.5 ± 0.1	0.4 ± 0.1
22:1n9	0.0 ± 0.0	0.0 ± 0.0	0.0 ± 0.0	0.0 ± 0.0	0.2 ± 0.0	0.0 ± 0.1
Others	1.5 ± 0.3	2.9 ± 0.6	4.4 ± 1.2	3.0 ± 0.2	4.9 ± 0.6	5.8 ± 0.6

Others include 16:1, 16:2, 24:0 and 24:1.

OA, Oleic acid; LA, linoleic acid; ALA, α‐linolenic acid; DGLA—dihomo‐γ‐linolenic acid; ARA, arachidonic acid; ETA, eicosatetraenoic acid; EPA—eicosapentaenoic acid.

As also observed for neutral lipids, the levels of 16:0 are markedly elevated in all three classes of phospholipid examined. In contrast, OA levels are very noticeably reduced, most pointedly in PC where the OA level is reduced to 15% of WT (5.8% versus 39%; Table 4). Similarly, LA levels are decreased in PC, PE and PI&PS, whereas in PC, ALA levels are effectively unchanged. As described above, some impact on the levels of OA and LA could be ascribed to the action of the PsΔ12‐desaturase, when working in conjunction with other activities to generate a flux. However, it is unclear as to why the levels of 16:0 are elevated, not least of all as this is known to be a product of the plastidial fatty acid synthase (FAS) and therefore not directly connected with extraplastidic lipid metabolism. It is possible that alterations to this latter pool are sensed and result in feedback to alter the activity of FAS—however, our previous lipidomic data from the expression of the conventional Δ6‐pathway in *C. sativa* did not reveal such a major alteration to the accumulation of 16:0, even though that engineering also resulted in the accumulation of EPA (Ruiz‐Lopez *et al*., 2014). Moreover, the fact that both configurations of the pathway resulted in greatly reduced levels of OA in PC strongly implicates the PsΔ12 in the extraplastidial aspect of this process.

### Triacylglycerol and phosphatidylcholine regio‐specific analyses in high EPA line expressing the alternative Δ9‐pathway

**Figure 3:**
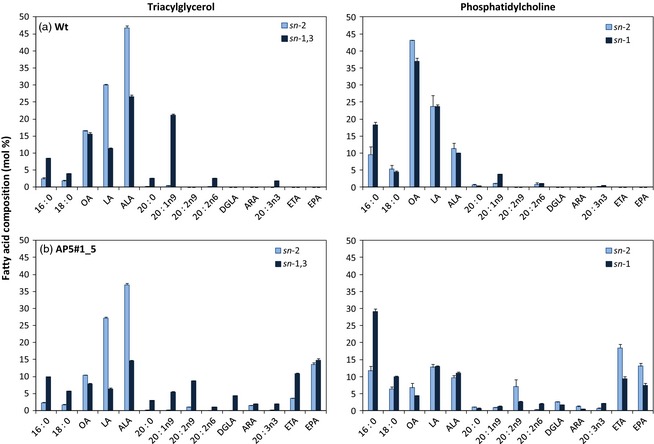
Stereospecific analysis of TAG and PC. The positional distribution of fatty acids in TAG (*sn*‐2 and *sn*‐1/3) and PC (*sn*‐1 and *sn*‐2) was determined in (a) wild‐type *Camelina sativa* and (b) AP5#1_5 (*n* = 3; ±SE).

To further define the assembly of EPA and DHA into seed oil, regio‐specific analyses were performed on the AP5_1_5 line, characterizing the distribution of EPA and DHA in both TAG and PC molecules. These analyses report an inherent bias of *C. sativa* acyltransferases with regard to their channelling of newly synthetized fatty acids such as ETA, 20:2*n*‐9, DGLA and ARA into the *sn*‐1,3 positions of seed TAGs (Figure 3), as previously observed before with the conventional Δ6‐pathway (Ruiz‐Lopez *et al*., 2014). Interestingly, EPA was equally distributed between the positions (*sn*‐1,3 and *sn*‐2) in TAG. This is similar to our previous observations for Camelina accumulating DHA, but distinct from the situation for EPA accumulation via the conventional pathway, where EPA was strongly enriched at the *sn*‐2 position of TAG. This difference may represent the combination of a number of different factors (such as acyl‐exchange and substrate channelling) represented schematically in Figure 2. In addition, we also found that endogenous 16:0, 18:0, 20:0, 20:1*n‐*9, 20:2*n‐*6 and 20:3*n‐*3 were biased towards the *sn‐*1,3 positions of TAG and other fatty acids (such as OA, LA and ALA) were biased towards the *sn*‐2 position of TAG, in agreement with previous observations.

Analysis of the distribution of fatty acids (*sn*‐1 versus *sn*‐2) on PC revealed that the two predominant nonnative fatty acids (ETA and EPA) were significantly enriched at the *sn*‐2 position, although it is interesting to note that ETA levels were higher than EPA, that is the substrate was greater than product. When this observation is taken into context with the differences in *sn*‐distribution in TAG for ETA versus EPA, these data provide more evidence for the substrate preference (acyl‐CoA as opposed to phospholipids) of the EhΔ5‐desaturase. Thus, ETA is generated via the phospholipid‐dependent PsΔ8‐desaturase, resulting in the accumulation of this fatty acid at the *sn*‐2 position of PC. The incorporation of ETA into TAG is then predominantly via the action of phospholipid: diacylglycerol acyltransferase (PDAT), resulting in the enrichment of ETA at the *sn*‐3 position of TAG. In contrast, EPA is generated by the Δ5‐desaturation of ETA‐CoA, requiring acyl‐exchange of ETA from PC into the acyl‐CoA pool prior to the action of the EhΔ5‐desaturase (Figure 2). The resulting product, EPA‐CoA, is then available for acylation via the Kennedy pathway, enabling EPA to accumulate at either the *sn*‐2 position [lysophosphatidic acid acyltransferase (LPAT)‐dependent acylation] or *sn*‐3 position [acyl‐CoA: diacylglycerol acyltransferase (DGAT)‐dependent acylation] of TAG. In that respect, it seems likely that the particular configuration of the alternative pathway used in the present study has inadvertently recreated the problem of substrate dichotomy, which is generally viewed as an issue for transgenic reconstitution of the conventional Δ6‐pathway (Sayanova and Napier, 2004). More specifically, the use of the acyl‐CoA‐dependent Δ5‐desaturase from *E. huxleyi* in conjunction with the preceding reaction of the phospholipid‐dependent Δ8‐desaturases (Figure 1a,b) means that acyl‐exchange of ETA from PC into the acyl‐CoA pool likely becomes rate‐limiting for the synthesis of EPA. However, as ETA is a naturally occurring component of omega‐3 fish oils, and itself an interesting fatty acid, our results demonstrate a novel approach for the synthesis of this compound.

In support of this, analysis of the acyl‐CoA pool in *C. sativa* developing seeds (WT and transgenic line AP5) revealed the addition of nonnative Δ9‐elongation products 20:2 and 20:3 in the acyl‐CoA pool, and a concomitant reduction in levels of 18:2‐CoA and 18:3‐CoA (reflecting the product–substrate relationship). Interestingly, the levels of 16:0‐CoA were strongly elevated in the AP5 developing seeds (17.0% versus 10.9% in WT); consistent with the suggestion above, that the overall increase in these fatty acids is mediated through increased activity of the plastidial FAS. However, it appears to be discrimination amongst the plastidially synthesized fatty acids (i.e. 16:0, 18:0 and 18:1) as the C18 forms are not altered in the acyl‐CoA between WT and AP5 seeds (Table 5). As mentioned above, the regulation of such a process remains obscure, but mechanistically it might be expected to involve the *FatB* C16 thioesterase. It should also be noted that these modulations in plastidial fatty acids appear to be distinct to that observed by Bates *et al*. (2014) on the feedback inhibition of FAS due to the presence of transgene‐derived hydroxylated fatty acids in glycerolipid‐based metabolism.

**Table 5:** Acyl‐CoA profiles in line AP5#1_5 (T3)

Analysis of Acyl‐CoA from *C. sativa* seed (23 DAF)		
Acyl‐CoA (Mol %)	Wild type	AP5#1_5
16:0	10.9 ± 1.7	17.0 ± 0.8
16:1	0.6 ± 0.1	0.3 ± 0.1
16:3	0.1 ± 0.1	0.2 ± 0.2
18:0	15.9 ± 4.0	17.6 ± 0.7
18:1	4.4 ± 0.6	5.6 ± 1.3
18:2	12.9 ± 2.6	7.4 ± 1.4
18:3	5.9 ± 0.6	4.8 ± 0.4
20:0	18.6 ± 1.3	17.2 ± 2.2
20:1	8.8 ± 3.8	2.4 ± 0.5
20:2	0.6 ± 0.3	5.8 ± 0.7
20:3	0.3 ± 0.1	4.0 ± 0.5
20:4	nd	3.4 ± 0.8
20:5	nd	2.4 ± 0.5
22:0	6.8 ± 1.3	6.0 ± 0.5
22:1	4.5 ± 1.7	1.4 ± 0.3
24:0	5.0 ± 1.8	2.3 ± 0.7
24:1	4.2 ± 2.2	2.3 ± 0.7

nd, Not detected; *C*. *sativa*,* Camelina sativa*.

### Incorporation of C20 omega‐3 fatty acids into TAG molecular species

**Figure 4:**
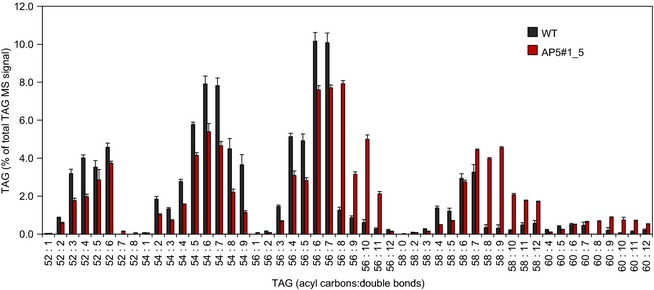
Analysis of major triacylglycerol species in wild‐type and engineered mature *Camelina sativa* seeds. TAG species were characterized using an ESI‐MS/MS neutral loss survey scan (*n* = 3; ±SE), with each TAG species represented by the total number of fatty acid carbon atoms:desaturations.

To better understand the accumulation of the target fatty acids within TAG, we performed LC‐MS/MS analyses to identify the individual molecular species of TAG present in mature seed oils of transgenic AP5_1_5 (T3 line). The three predominant TAGs in WT were 56:6 (10.2%), 56:7 (10.1%) and 54:6 (7.9%) (Figure 4). However, in the AP5 line, there are a number of additional TAG species, including several which accumulate to the levels similar to the most abundant native species. For example, the TAG species 56:8 represents 7.9% of the total TAG species identified by our survey—this TAG most likely represents EPA+18:1 + 18:1. Other abundant candidate species for mono‐EPA TAGs include 56:10 (EPA+18:3 + 18:2) and 56:11 (EPA+18:3 + 18:3), and it is highly likely that species in the range 58:10‐58:12 represent TAGs which contain two molecules of EPA (e.g. 58:10 = EPA + EPA + 18:0) (Figure 4). No TAG species with a mass equivalent to tri‐EPA (or tri‐ETA) were detected, although TAGs which likely contained 3 C20 polyunsaturated fatty acids were observed at low levels (e.g. 60:12). Thus, it is clear that nonnative C20 omega‐3 polyunsaturated fatty acids are actively incorporated into TAGs in Camelina, confirming previous observations and also indicating the presence of useful endogenous acyltransferases with which to enable this metabolic engineering.

## Conclusions

In conclusion, the accumulation of high levels of LC‐PUFAs, specially EPA and/or DHA in plant oils is an important goal of the metabolic engineering community. In this study, we have efficaciously bypassed the previously reported bottlenecks in the pathway, and as a result, we have successfully reconstituted an alternative pathway for the biosynthesis and accumulation of EPA in the seeds of an oilseed crop, *C. sativa*. However, our results also highlight the importance of knowledge‐directed engineering. Given that our understanding of plant lipid metabolism is still expanding, the challenge still remains to deliver predictive modifications to seed oil composition (Figure 2). In addition, our transgenic Camelina AP5 lines represent a new resource for the evaluation of the role of the marine fatty acid ETA in omega‐3 dietary metabolism and nutrition (Figure S3).

## Experimental procedures

### Plant material and growth conditions

Plants of *A. thaliana and C. sativa* were grown for analyses in a controlled environment chambers at 23 °C day/18 °C night, 50%–60% humidity, and kept on a 16‐h, 250 μmol/m^2^/s, photoperiod (long day).

### Generation of transgenic plants

Transgenic Arabidopsis lines were generated as previously described (Sayanova *et al*., 2006a,b), whilst transgenic *C. sativa* lines were generated as previously described with minor modifications (Ruiz‐Lopez *et al*., 2014). The designed vectors were transferred into *Agrobacterium tumefaciens* strain AGL1. *C. sativa* inflorescences were immersed in the Agrobacterium suspension for 30 s without applying any vacuum. Transgenic seeds were identified on the basis of resistance to kanamycin, conferred by the *NPTII* transgene. In all cases, no phenotypic perturbation was observed as a result of modification of the seed oil composition.

### Vector construction

Four constructs, AP2, AP3, AP4 and AP5, were built using the Gateway^®^ recombination system (Invitrogen, Paisley, UK) as previously described in Ruiz‐López *et al*. (2012). Respective genes were inserted as NcoI/PacI or Asc/PacI fragments into the promoter/terminator cassettes and then moved into pENTRY vectors. All open‐reading frames for desaturases and elongases were resynthesized (GenScript Corporation, Piscataway, NJ) and codon‐optimized for expression for *C. sativa*. The destination vector contained an *NPTII* gene as the selection marker, driven by the constitutive *nos* promoter.

The gene construct, AP2 (Figure 1b), comprised of five expression cassettes, including (i) Cnl, a conlinin 1 promoter (Truksa *et al*., 2003), EhElo9, a Δ9‐elongase from *Emiliania huxleyi* (Petrie *et al*., 2010), and OCS, a terminator region of OCS, octopin synthase gene of *Agrobacterium tumefaciens*; (ii) Cnl promoter, EhΔ8, a Δ8‐desaturase from *E. huxleyi* (Sayanova *et al*., 2011b) and the OCS terminator; (iii) Cln promoter, EhΔ5, a Δ5‐desaturase from *E. huxleyi* (Sayanova *et al*., 2011b) and the OCS terminator; (iv) Np, a *Brassica napus* napin promoter, PsΔ12, a Δ12‐desaturase gene from *Phytophthora sojae* (Cirpus and Bauer, 2006), and E9, a terminator of pea rbcS‐E9; (v) Np promoter, Piω3, a ω3‐desaturase gene from *Phytophthora infestans* (Wu *et al*., 2005), and E9 terminator.

A similar approach was used to build the other five gene constructs. AP3, included EhΔ8, a Δ8‐desaturase from *Acanthamoeba castellanii* (Sayanova *et al*., 2006a,b), instead of EhΔ8. AP4 included IsElo9, a Δ9‐elongase gene from *I. galbana* (Qi *et al*., 2002) instead of EhElo9 and PsΔ8, a Δ8‐desaturase from *P. salina* (Zhou *et al*., 2007) instead of AcΔ8 in construct AP2. Both genes were under control of the *Phaseolus vulgaris* PvArc promoter and linked to the PvArc terminator region, as previous described (Ruiz‐Lopez *et al*., 2013). Finally, AP5 construct included EhElo9, IsElo9, PsΔ8, EhΔ5, Piω3 and PsΔ12 genes.

### Fatty acid analysis

Total fatty acids in seed batches were extracted and methylated by heating the samples at 80 °C for 2 h with 2 mL of a solution containing methanol/toluene/dimethoxypropane/H_2_SO_4_ (66:28:2:1 by volume) as previously described (Garces and Mancha, 1993). After cooling, 1.5 mL of hexane was added, and fatty acid methyl esters were recovered from the upper phase. Methyl ester derivatives of total fatty acids extracted were analysed by GC‐FID (flame ionization detection) using an Agilent 6890 gas chromatography system (Palo Alto, CA) with a AT‐225 capillary column of fused silica (30 m length, 0.25 mm i.d., 0.20 μm film thickness). Hydrogen was used as the carrier gas. Fatty acids were identified by comparison with known standards (Sigma, St. Louis, MO), and they were confirmed by GC‐MS. Values presented are representative numbers derived from replicated analyses.

### Lipid extraction and separation

Three hundred milligrams of seeds was heated for 10 min at 95 °C in 1 mL isopropanol and homogenized using a mortar and pestle. The homogenate was centrifuged at 3000 g for 15 min at room temperature, supernatant was collected, and the pellet was re‐extracted with isopropanol/chloroform (1:1 v/v). Both extracts were pooled, evaporated, and dissolved in chloroform/acetic acid (100:1 v/v). The lipid extract was loaded on a Sep‐Pak column (www.waters.com) and prefractionated into neutral lipids, glycolipids and phospholipids by adding chloroform/acetic acid (100:1 v/v), acetone/acetic acid (100:1 v/v) and methanol, respectively. These fractions were further resolved on TLC silica gel plates (thickness 0.25 mm). Neutral lipids were developed using hexane/ethyl ether/formic acid (75:25:1 by volume), and polar lipids were developed using chloroform/methanol/ammonia/water (70:30:4:1 by volume). The individual lipid classes were identified under UV light after spraying with primuline (0.05% w/v in acetone/water, 80:20 v/v), scraped from the plate, and used directly for methylation or extracted for further analysis.

### Positional analysis of TAG and PC

Positional analysis of purified TAG was performed as described previously by Luddy *et al*. (1964). Samples containing 5 mg TAG were dried under nitrogen and resuspended in 1 mL of 1 mm Tris‐HCl (pH 8.0). Samples were then sonicated for 60 s to ensure complete emulsification of the lipid. Then, 0.1 mL of 22% CaCl_2_ and 0.25 mL of 0.1% deoxycholate were added. Samples were warmed at 40 °C for 30 s, and 2 mg pancreatic lipase (≥20 000 units per mg protein; Sigma) was added. Samples were vortexed for 2–3 min. The reaction was terminated using 0.5 mL 6 m HCl. The lipids were extracted twice with 2.5 mL diethyl ether. Lipids were evaporated at 40 °C under nitrogen and separated into lipid classes by TLC using silica plates and hexane/diethyl ether/acetic acid (70:30:1 by volume). The spots corresponding to 2‐monoacylglycerols were scraped from the plate and directly transmethylated for GC‐FID analysis. The mean composition of fatty acids in the *sn*‐1,3 positions was calculated using the composition of an aliquot of the initial triacylglycerol and the formula: mean percentage *sn*‐1,3 = [(3 × % fatty acid in triacylglycerol) — (% fatty acid in the *sn*‐2 position)]/2.

Positional analysis of purified PC was performed as described previously (Ruiz‐Lopez *et al*., 2009). Briefly, analysis of PC was performed using *Naja mossambica* phospholipase A2 (Sigma, St. Louis, MO). Samples containing PC were dried under nitrogen and resuspended in 1 mL borate buffer (0.5 m, pH 7.5, containing 0.4 mm CaCl_2_) by sonication. Five units of lipase and 2 mL diethyl ether were added, and the digestions were performed for 2 h. The ether phase was evaporated, and the reaction was stopped by adding 0.3 mL of 1 m HCl. The aqueous phase was extracted using chloroform/methanol (2:1 v/v). The resulting organic phase was dried under nitrogen and separated by TLC using chloroform/methanol/aqueous ammonia (65:25:0.7 v/v/v) as the solvent mix. The spots corresponding to free fatty acids and lysophospholipids were scraped from the plate and directly transmethylated for GC‐FID analysis.

### Acyl‐CoA analyses

Freshly harvested seeds were frozen in liquid nitrogen, and acyl‐CoAs were extracted as described by Larson and Graham (2001) and analysed using LC‐MS/MS + MRM in positive ion mode. The LC‐MS/MS + MRM analysis (using an ABSciex 4000 QTRAP Framingham, MA) was performed as described by Haynes *et al*. (2008), (Agilent 1200 LC system; Gemini C18 column (Phenomenex, Torrance, CA), 2 mm inner diameter, 150 mm length, particle size 5 μm). For the identification and calibration, standard acyl‐CoA esters with acyl chain lengths from C14 to C20 were purchased from Sigma as free acids or lithium salts.

### Polar lipid analyses

Lipids were extracted from *C. sativa* seed and analysed by ESI‐MS/MS using methods adapted from Lee *et al*. (2011). To extract lipids from mature seed, five seeds were smashed and transferred immediately to 3 mL of hot isopropanol and extracted as described above. The extract was washed with 1 mL of 1 m KCl and then 2 mL water. The solvent was evaporated under nitrogen, and the dry lipid extract was dissolved in 1 mL of chloroform. The molecular species of polar lipids were analysed by ESI triple‐quadrupole mass spectrometry (API 4000; Applied Biosystems, Paisley, UK). The molecular species of polar lipid were defined by the presence of a head‐group fragment and the mass/charge of the intact lipid ion formed by ESI. Such tandem ESI‐MS/MS precursor and product ion scanning, based on head‐group fragment, do not determine the individual fatty acyl species. Instead, polar lipids are identified at the level of class, total acyl carbons and total number of acyl carbon–carbon double bonds. Polar lipids were normalized by comparing a series of polar lipid internal standards and expressed as a total percentage of the MS peak area signal.

### Profiling triacylglycerol composition

The molecular species of TAGs were analysed by electrospray ionization triple‐quadrupole mass spectrometry (API 4000 QTRAP; Applied Biosystems). Triacylglycerols were measured after Krank *et al*. (2007) and were defined by the presence of one acyl fragment and the mass/charge of the ion formed from the intact lipid (neutral loss profiling). This allows identification of one TAG acyl species and the total acyl carbons and total number of acyl double bonds in the other two chains. The procedure does not allow identification of the other two fatty acids individually nor the positions (*sn*‐1, *sn*‐2, or *sn*‐3) that individual acyl chains occupy on the glycerol. TAGs were quantified after background subtraction, smoothing, integration, isotope deconvolution and comparison of sample peaks with those of the internal standard (using LipidView™; Applied Biosystems). The profiling samples were prepared by combing 50 μL of the total lipid extract with 950 μL of isopropanol/methanol/50 mm ammonium acetate/dichloromethane (4:3:2:1). Samples were infused at 15 μL/min with an autosampler (LC mini PAL, CTC Analytics, Switzerland, Paisley, UK). The scan speed was 100 μ/s. The collision energy, with nitrogen in the collision cell, was +25 V; declustering potential was +100 V; entrance potential was 14 V; and exit potential was +14 V. Sixty continuum scans were averaged in the multiple channel analyser mode. For product ion analysis, the first quadrupole mass spectrometer (Q1) was set to select the TAG mass and Q3 for the detection of fragments fragmented by collision induced dissociation. The mass spectral responses of various TAG species are variable, owing to differential ionization of individual molecular TAG species. For all analyses, gas pressure was set on ‘low’, and the mass analysers were adjusted to a resolution of 0.7 μ full width height. The source temperature was 100 °C; the interface heater was on, and +5.5 kV was applied to the electrospray capillary; the curtain gas was set at 20 (arbitrary units; and the two ion source gases were set at 45 (arbitrary units). In the data shown herein, no response corrections were applied to the data. The data were normalized to the internal standards tri15:0 and tri19:0 (Nu‐Chek Prep, Elysian, MN).

## Supporting information


**Figure S1** Differentiation of ETA from ARA and juniperonic acid.
**Figure S2** Range of ETA and EPA content in single seeds from AP5#1_5.
**Figure S3** Comparison of fatty profiles for fish oil, *Camelina* and AP5#1_5.

**Table S1** Single seed analysis – fatty acid composition of individual seeds of line AP5#1_5.
