# Supplementary material for: An alternative pathway for the effective production of the omega‐3 long‐chain polyunsaturates EPA and ETA in transgenic oilseeds
Source: Plant Biotechnol J. 2015 Jan 30;13(9):1264–75. doi: 10.1111/pbi.12328 (PMC4973703; doi:10.1111/pbi.12328)
Supplement: Supplementary file 1 — Figure S1 Differentiation of ETA from ARA and juniperonic acid. Figure S2 Range of ETA and EPA content in single seeds from AP5#1_5. Figure S3 Comparison of fatty profiles for fish oil, Camelina and AP5#1_5. [file PBI-13-1264-s001.pptx]

## Slide 1
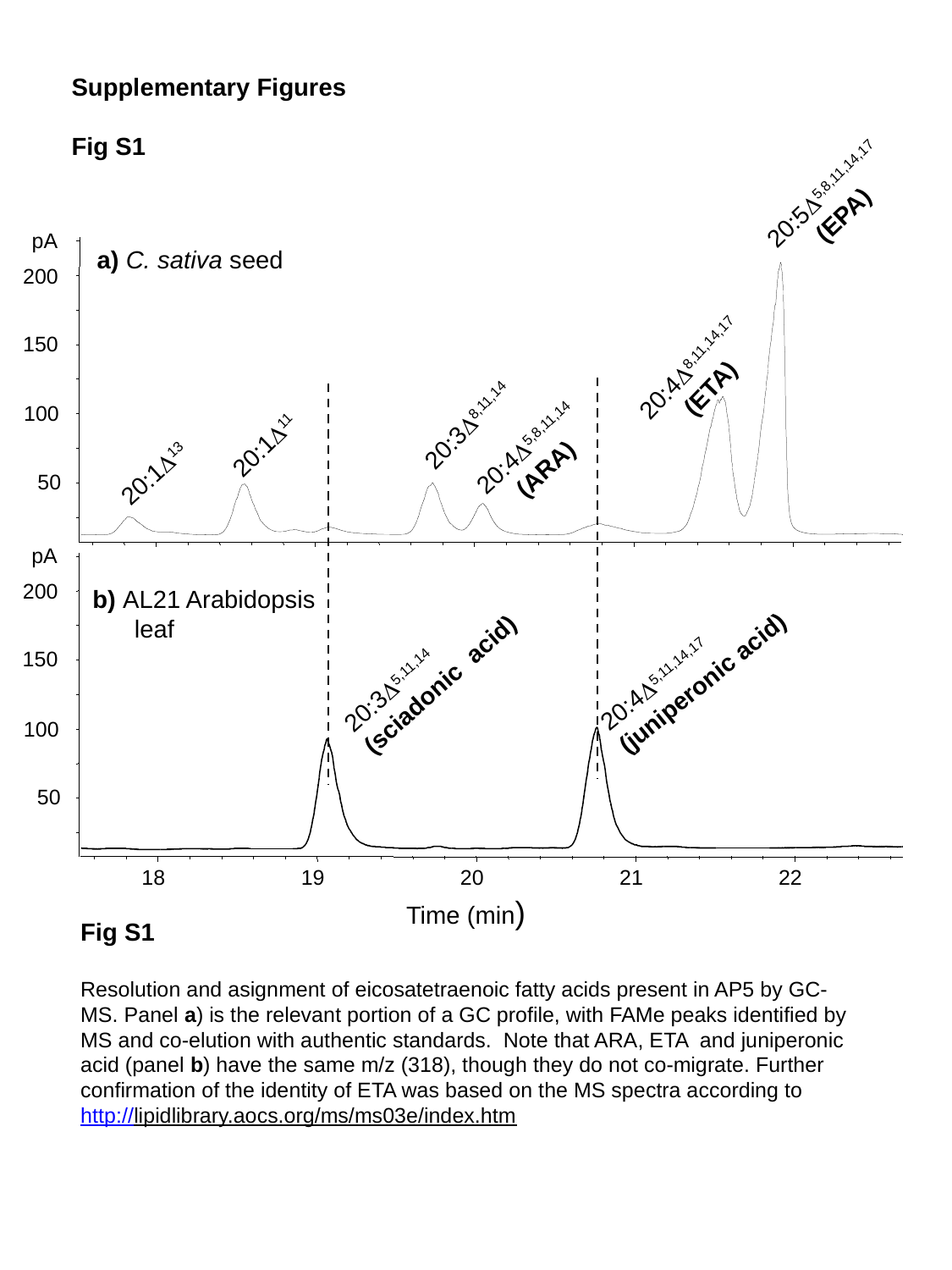

Supplementary Figures
Fig S1
20:5D5,8,11,14,17
(EPA)
pA
200
150
100
50
a) C. sativa seed
20:4D8,11,14,17
(ETA)
20:3D8,11,14
20:4D5,8,11,14
(ARA)
20:1D11
20:1D13
pA
200
150
100
50
b) AL21 Arabidopsis
 leaf
20:4D5,11,14,17
(juniperonic acid)
20:3D5,11,14
(sciadonic acid)
18
19
20
21
22
Time (min)
Fig S1
Resolution and asignment of eicosatetraenoic fatty acids present in AP5 by GC-MS. Panel a) is the relevant portion of a GC profile, with FAMe peaks identified by MS and co-elution with authentic standards. Note that ARA, ETA and juniperonic acid (panel b) have the same m/z (318), though they do not co-migrate. Further confirmation of the identity of ETA was based on the MS spectra according to http://lipidlibrary.aocs.org/ms/ms03e/index.htm

## Slide 2
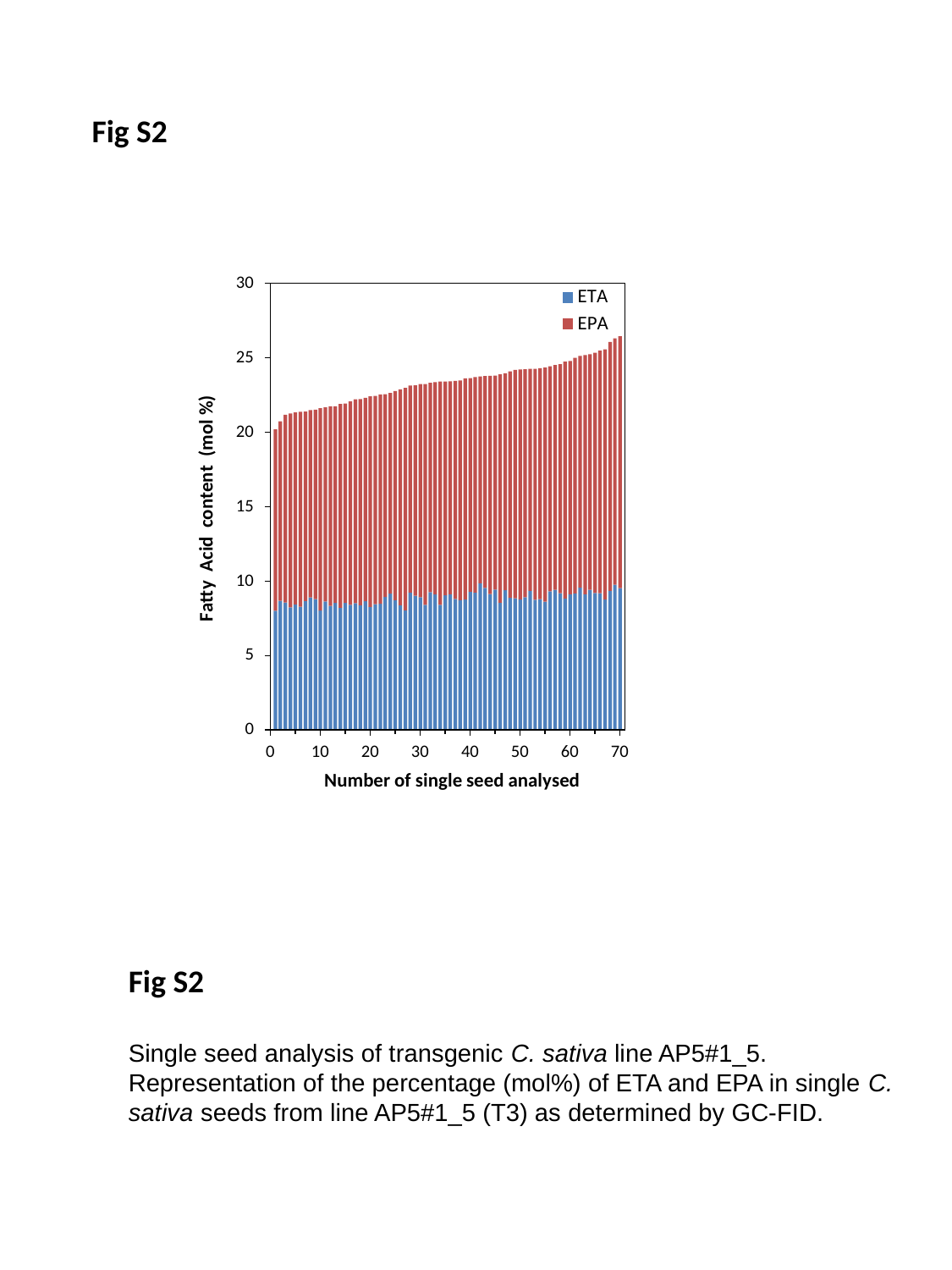

Fig S2
Fig S2
Single seed analysis of transgenic C. sativa line AP5#1_5. Representation of the percentage (mol%) of ETA and EPA in single C. sativa seeds from line AP5#1_5 (T3) as determined by GC-FID.

## Slide 3
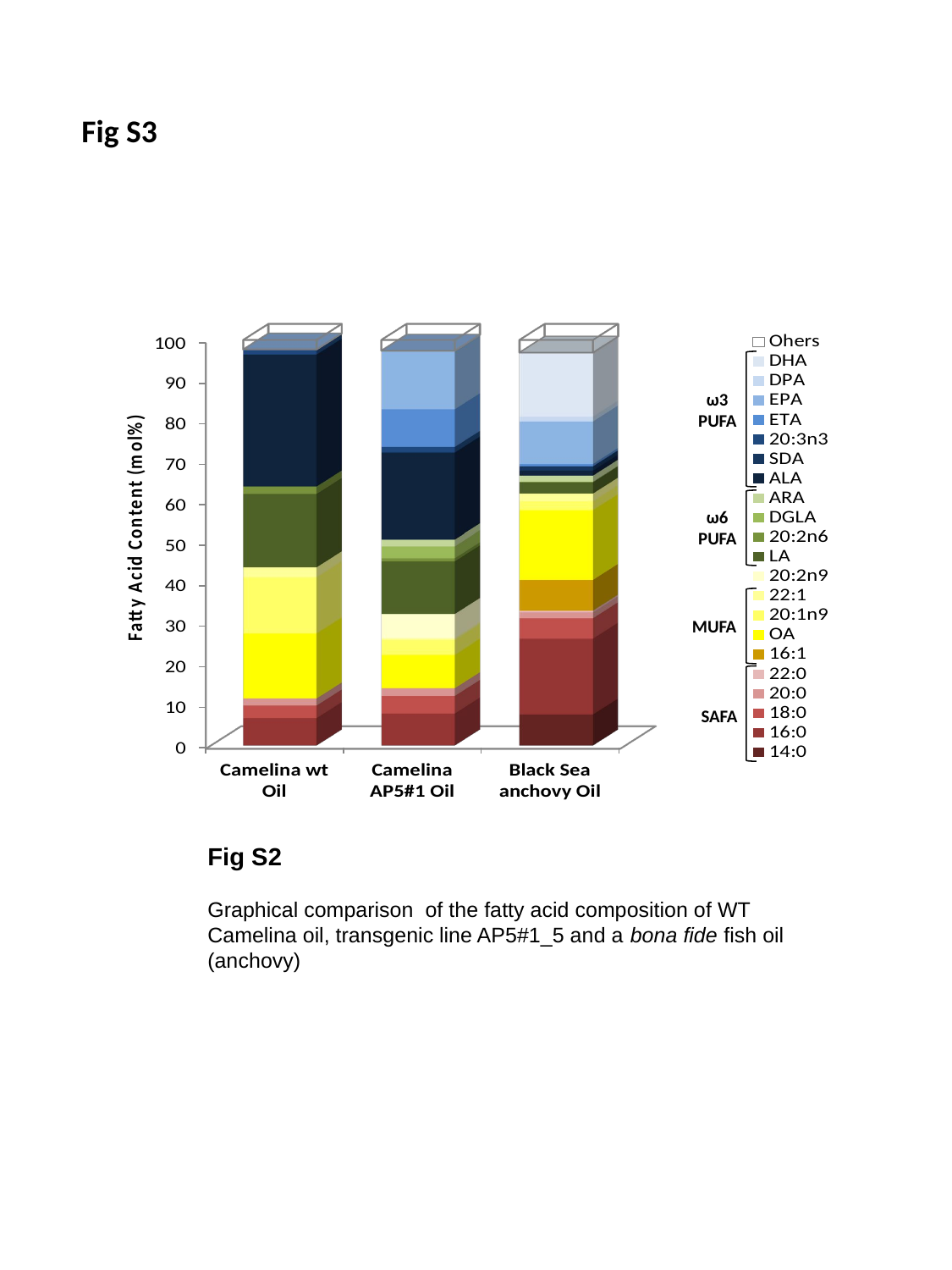

Fig S3
ω3
PUFA
ω6
PUFA
MUFA
SAFA
Fig S2
Graphical comparison of the fatty acid composition of WT Camelina oil, transgenic line AP5#1_5 and a bona fide fish oil (anchovy)
